# Validation of a Highly Specific Anti-Human Folate Receptor Alpha Single-Domain Antibody for Rapid Fluorescence-Guided Surgery of Ovarian and Lung Cancer

**DOI:** 10.3390/biom16101426

**Published:** 2026-10-01

**Authors:** Sam Floru, Stijn Vandorpe, Laurent Navarro, Shivakrishna Kallepu, Madduri Srinivasarao, Philip Stewart Low, Matthias D’Huyvetter, Sophie Hernot

**Affiliations:** 1 Molecular Imaging and Therapy Research Group (MITH), Faculty of Medicine and Pharmacy, Brussels Health Campus, Vrije Universiteit Brussel (VUB), Laarbeeklaan 103, 1090 Brussels, Belgium; sam.floru@vub.be (S.F.); stijn.vandorpe@gmail.com (S.V.); matthias.dhuyvetter@vub.be (M.D.); 2 Precirix NV, 1090 Brussels, Belgium; laurent.n@hotmail.com; 3 Institute for Drug Discovery, Purdue University, West Lafayette, IN 47907, USA; shivakrishna.kallepu@moffitt.org (S.K.); msriniva@purdue.edu (M.S.); plow@purdue.edu (P.S.L.)

**Keywords:** fluorescence-guided surgery, single-domain antibody, OTL-38, human folate receptor, human folate receptor alpha, ovarian cancer, lung cancer

## Abstract

Despite the increasing clinical adoption of molecular fluorescence-guided surgery (FGS), the limited tumour specificity of currently available contrast agents remains a challenge for accurate tumour identification. This study aimed to validate a single-domain antibody (sdAb)-based tracer specifically targeting the human folate receptor alpha (hFRα) and evaluate its specificity and in vivo pharmacokinetics compared to an established folate receptor targeted tracer. The anti-hFRα sdAb 2BD42 was conjugated to the near-infrared dye s775z. Following quality control, in vitro studies assessed binding specificity towards hFRα and hFRβ. Subsequently, its longitudinal in vivo biodistribution profile in a subcutaneous xenograft model was compared to that of OTL-38. Finally, the applicability of sdAb 2BD42-s775z was evaluated as a proof-of-concept in ovarian carcinomatosis and orthotopic lung tumour models. Following successful fluorescence labelling, sdAb 2BD42-s775z retained sub-nanomolar affinity for hFRα and selectively discriminated hFRα from hFRβ in vitro, in contrast to OTL-38. Furthermore, sdAb 2BD42-s775z exhibited a rapid tumour accumulation and a relevant tumour-to-background ratio (TBR) of 4.1 ± 0.9 at 1 h post-injection which persisted for up to 1 week post-injection, while for OTL-38, a relevant TBR was developed more slowly from 24 h post-injection onwards. In addition, sdAb 2BD42-s775z was able to rapidly and specifically highlight tumour lesions in an ovarian carcinomatosis and orthotopic lung tumour model, whereas no tumour uptake was observed with an irrelevant sdAb. To conclude, 2BD42-s775z demonstrated high specificity for hFRα, favourable TBR contrast, and rapid tumour visualisation in preclinical ovarian and lung cancer models, highlighting its potential for FGS. Further studies are warranted to determine whether selective hFRα targeting improves the detection of lymph node metastases compared with currently available folate receptor-targeted tracers.

## 1. Introduction

The treatment approach for solid tumours largely depends on the stage at which the cancer is diagnosed. In patients with early-stage disease, treatment often includes curative-intent surgery, either alone or in combination with neoadjuvant and/or adjuvant systemic therapy or radiotherapy, with the goal of achieving complete tumour eradication while preserving healthy tissue and organ function whenever possible [1]. In more advanced disease, surgery may still play an important role, either with curative intent in selected patients with resectable locally advanced or oligometastatic disease or as debulking (cytoreductive) surgery to reduce tumour burden when complete resection is not feasible (e.g., late-stage ovarian cancer), thereby facilitating other treatment modalities and improving disease control [1,2]. To improve surgical precision, fluorescence-guided surgery (FGS) with targeted or activatable contrast agents such as pafolacianine (Cytalux™) and pegulicianine (Lumisight™) serves as an intraoperative imaging tool to enhance the visualisation of the tumour to obtain a more complete tumour resection or a maximal cytoreduction [3,4,5].

Cytalux™ (pafolacianine, OTL-38), a small molecule targeting the human folate receptor (hFR) which has elevated expression levels on various cancer types [6,7,8,9], gained clinical approval from the FDA in 2021 for the identification of ovarian cancerous lesions during debulking surgery [10]. During a phase 3 clinical trial, in 39.7% of patients undergoing interval debulking surgery, Cytalux™ enabled the identification of additional ovarian cancer lesions not planned for resection and not detected by palpation and white light assessment, showcasing its important real-time value during surgery [11]. In 2022, it also received approval from the FDA for the visualisation of lung cancer during surgery (NCT04241315) [12,13]. Currently, a phase 4 dose extension study is recruiting patients with ovarian and lung cancer to evaluate different injection-to-imaging intervals for FGS (NCT07469202) [14]. In addition, to extend the scope of clinical applications, clinical trials are currently being conducted to assess the safety and efficacy of the contrast agent in prostate and gastrointestinal malignancies (NCT06434909; NCT07124351) [15,16]. However, false positive signals have been observed using Cytalux™ due to the recognition of not only human folate receptor alpha (hFRα) but also beta (hFRβ). Hence, the expression of hFRβ on activated anti-inflammatory macrophages residing in the lymph nodes may complicate the detection of lymph node metastasis [17].

Single-domain antibodies (sdAbs or also often referred to as Nanobodies or VHHs), which are derived from heavy-chain only antibodies from *Camelids*, have already proven to be promising alternative targeting moieties for FGS [18,19,20]. sdAbs have a small size (12–15 kDa) with a robust structure, a high in vivo stability, solubility, and affinity for their target. This enables a short injection-to-imaging time interval and highly-specific imaging [21,22,23,24,25]. Additionally, due to a high sequence similarity with human IgG, sdAbs have a low immunogenicity in humans [26]. As compared to small molecules and peptides, the sdAb platform technology allows generation of targeting moieties against virtually any target of interest, with predictable pharmacokinetics.

An anti-hFRα sdAb named 2BD42 was previously generated by Dewulf et al. When radiolabelled with ^18^F, this sdAb enables high uptake in hFRα-expressing tumours in mice as early as 1 h post-injection with low uptake in healthy organs [27], thereby supporting its potential for PET imaging. Furthermore, its ^131^I-labelled version demonstrated therapeutic efficacy in tumour-bearing mice, resulting in prolonged survival without observable toxicity [28].

In this study, we aimed to develop sdAb 2BD42 as a fluorescent tracer for FGS, providing selective hFRα targeting and a more favourable injection-to-imaging time interval as compared to Cytalux™. To do so, the sdAb was labelled with the near-infrared (NIR) dye s775z. Subsequently, the biodistribution and in vivo tumour targeting was determined through fluorescence imaging and compared to the in vivo pharmacokinetic properties of OTL-38. Finally, this study aimed to establish a proof-of-concept (PoC) for two clinical applications of FGS in an ovarian carcinomatosis tumour model and an orthotopic lung cancer model.

## 2. Materials and Methods

### 2.1. Fluorescence Labelling of sdAb 2BD42 and Control sdAb R3B23

A total of 3 mg of untagged anti-hFRα sdAb 2BD42 [28] and control sdAb R3B23 [29] were separately buffer-exchanged from 1x phosphate-buffered saline (PBS) into 450 µL 500 mM borate buffer (pH = 8.7) using a Vivaspin^®^ 2 (5000 MWCO HY, Sartorius, UK) centrifugal concentrator at 2000× *g*. Following buffer exchange, sdAb 2BD42 and sdAb R3B23 were incubated with 3.5 molar excess of NHS-ester-activated fluorescent dye s775z (Fluoroprobes, Scottsdale, AZ, USA) for a period of 90 min at 21 °C in the dark. Subsequently, the purification of the fluorescently labelled sdAbs was performed by size-exclusion chromatography (NGCTM Chromatography System, Bio-Rad, Hercules, CA, USA) using a Superdex™ 30 Increase 10/300 GL column (Cytiva, Marlborough, MA, USA) with 1x PBS as elution buffer at a flow rate of 0.8 mL/min. The labelled sdAbs were collected in different fractions of 500 µL by a BioFrac™ fraction collector (Bio-Rad). The concentration of the dye (C_dye_) and the sdAbs (C_sdAb_) was determined by measuring the absorbance at wavelengths 775 nm and 280 nm, respectively, using a UV–Vis spectrophotometer (NanoDrop™ 2000, Thermo Fisher Scientific, Waltham, MA, USA) and the Lambert–Beer law. A correction factor was applied to the dye’s absorbance at 280 nm by subtracting 3% of the absorbance measured at 775 nm from the value measured at 280 nm. A degree of labelling (DOL) of 1 was aimed for as this is the optimal DOL to obtain sufficient fluorescence signal and favourable in vivo tumour-to-background ratios, and to preserve the biological activity of the sdAb [30].

### 2.2. Quality Control of sdAb 2BD42-s775z

The purity of fluorescently-labelled sdAb 2BD42-s775z was first assessed using size-exclusion chromatography (SEC). To this end, 100 µg was loaded onto a Superdex™ 30 Increase 10/300 GL column (Cytiva) with 1x PBS as elution buffer at a flow rate of 0.8 mL/min. The area under the curve of the different peaks of interest was measured to determine the percentage of impurity in the sample. In addition, SDS-PAGE was performed. Here, 5 µg of both the unlabelled and the fluorescently labelled sdAb 2BD42 were mixed with non-reducing Laemmli buffer (2X; Bio-Rad). Following incubation at 95 °C for 4 min, the samples were loaded on a 16% Tris-Glycine Gel (Novex™ Wedgewell™, Life Technologies, Waltham, MA, USA) and run for 10 min at 80 V followed by 65 min at 150 V. Visualisation of the proteins on the gel was performed by incubation of the gel in Instant Blue for 30 min followed by 2 washing steps in MilliQ water. Next, gels were scanned with the Amersham™ Imager 680RGB (General Electric, Cincinnati, OH, USA) and the Odyssey fluorescence scanner (LI-COR Biosciences, Lincoln, NE, USA).

The mean molecular weight of the fluorescently labelled sdAb 2BD42-s775z was determined by electrospray ionization quadrupole time-of-flight (ESI-Q-TOF-MS; Orbitrap Biopharma platform) at a concentration of 0.1 µg/µL. The output mass range was set between 10 and 20 kDa. ESI-Q-TOF-MS was performed by Vlaams Instituut voor Biotechnologie (VIB; Ghent, Belgium).

The spectral characteristics of sdAb 2BD42-s775z were determined by transferring 1 µM of the tracer in quartz cuvettes. Excitation and emission spectra were acquired using a spectrofluorometer RF-6000 (Shimadzu, Kyoto, Japan) scanning from 200 to 900 nm at 1 nm intervals with a scan speed of 2000 nm/min. Data was normalised to the highest value obtained.

The tracer (C_tracer_ around 1 mg/mL) was subjected to stability studies at 21 °C in 1x PBS over 24 h (0 h, 1 h, 3 h and 24 h), while shielded from light. Additionally, tracer stability in serum and its potential binding to serum proteins were evaluated by incubating the samples in human serum (Merck Life Science, Darmstadt, Germany) at 37 °C for 1 h. Subsequently, samples were analysed by SEC (Hitachi Chromaster; Tokyo, Japan) using a Superdex™ 75 Increase 5/150 GL column (Cytiva) with 1x PBS as elution buffer at a flow rate of 0.3 mL/min.

To assess the affinity of sdAb 2BD42 towards the hFRα receptor after fluorescent labelling with the dye s775z, the k_a_, k_d_, and K_D_ of sdAb 2BD42 and sdAb 2BD42-s775z were compared by surface plasmon resonance (SPR) using a Biacore 1K+ (Cytiva, Marlborough, MA, USA). Here, a carboxylic sensor chip (Biacore Series S Sensor Chip CM5; Cytiva) was activated via 0.4 M/0.1 M 1-ethyl-3-(3-dimethylaminopropyl)carbodiimide/N-hydroxysuccinimide chemistry (EDC/NHS; Amine coupling Kit; Cytiva) in HBS buffer (10 mM Hepes, 150 mM NaCl, 0.005% Tween-20, pH 7.4). Following subsequent immobilisation of human hFRα recombinant protein (FOLR1; Sino Biological, Beijing, China) in 10 mM sodium acetate (NaOAc) pH 5.5, unbound hFRα was washed away with 50 nM NaOH, and unbound NHS-ester was deactivated using 1 M ethanolamine pH 8.5 (Amine Coupling kit; Cytiva). The requested immobilisation level was set at 190 RU. Subsequently, the samples were analysed in a two-fold serial dilution in HBS (100 nM–0.10 nM) with an analyte flow rate of 30 µL/min. The association and dissociation phase took 410 s and 900 s, respectively. Regeneration was carried out by injection of 50 mM glycine-HCl, pH 2.5, 2 M MgCl_2_ for 30 s at a flow rate of 30 µL/min, followed by a stabilisation phase of 120 s. Binding curves were fitted using a ‘1:1 (antigen–analyte)’ binding model in Biacore™ Insight Evaluation Software Version 6.0.7.1750.

### 2.3. Cell Lines and Culture Conditions

Human ovarian carcinoma cells (SKOV-3) were obtained from American Type Culture Collection (ATCC, Manassas, VA, USA). SKOV-3 cells were either used as received or transduced with Firefly Luciferase (FLuc). Both cell lines were cultured in McCoy’s 5A (modified) medium (Gibco™, Life Technologies, Waltham, MA, USA). Furthermore, murine hFRα-overexpressing TC-1 FLuc^+^ lung cancer cells were cultured in RPMI 1640 medium (Gibco™, Life Technologies). The transfer plasmid pDUAL-FLuc/PuroR encoding the FLuc and a puromycin resistance (PuroR) gene were generated using standard cloning techniques [31], and lentiviral transduction was performed as previously described [32]. Puromycin was used to select FLuc^+^ cells at a final concentration of 1 µg/mL. All media were supplemented with 10% fetal bovine serum (FBS; PAN-Biotech GmbH, Aidenbach, Germany), 1% L-glutamine (200 mM, 100x; Gibco™, Life Technologies), 1% penicillin (10,000 units/mL)/streptomycin (10,000 µg/mL; Gibco™, Life technologies). Cells were incubated at 37 °C in atmosphere with 5% CO_2_. After 80–90% confluency was reached, cells were split 1/5 following dissociation with TrypLE™ Express Enzyme (1x, Gibco™, Life Technologies). All cells were confirmed to be Mycoplasma-negative and checked for other relevant pathogens. Both cell lines SKOV-3 and TC-1 hFRα^+^ were previously validated and confirmed to express hFRα [27,33]. Human immortalised monocytic cells (THP-1) were obtained from ATCC. Cells were cultured in RPMI medium (Gibco™, Life Technologies) supplemented with 10% fetal bovine serum (FBS; PAN-Biotech GmbH), 1% L-glutamine (200 mM, 100x; Gibco™, Life Technologies), 1% penicillin (10,000 units/mL)/streptomycin (10,000 µg/mL; Gibco™, Life Technologies), and 0.05 mM 2-mercaptoethanol (Merck Life Science). Cells were split 1/5 twice a week.

### 2.4. In Vitro Validation of sdAb 2BD42-s775z

A cell binding study was performed to assess the tracer’s specific binding to hFRα-expressing cells, in comparison to OTL-38 (kindly provided by Prof. Philip Stewart Low, Purdue University, West-Lafayette, IN, USA). To do so, SKOV-3 cells were scraped using a 39 cm Cell Scraper (VWR, Leuven, Belgium). Hereafter, 400,000 cells were incubated for 1 h at 4 °C with 100 nM of fluorescently labelled sdAb 2BD42-s775z, OTL-38, or the irrelevant control sdAb R3B23-s775z. Subsequently, samples were washed 3 times with ice cold 1x PBS and transferred to a 24-well plate (Greiner bio-one, Frickenhausen, Germany). Finally, the 24-well plate was scanned with the Odyssey fluorescence scanner (LI-COR biosciences) using the 800 nm channel. In addition, a cell binding study was performed to confirm that sdAb 2BD42-s775z does not recognise the hFRβ and was again compared to OTL-38 and sdAb R3B23-s775z. A total of 750,000 hFRβ-expressing THP-1 cells were incubated for 1 h at 4 °C with 100 nM of sdAb 2BD42-s775z, OTL-38, or the negative control sdAb R3B23-s775z. Samples were washed 3 times with ice-cold 1x PBS and transferred into 24-well plates (Greiner bio-one). Finally, the plates were scanned with the Odyssey fluorescence scanner (LI-COR biosciences) using the 800 nm channel.

### 2.5. Animal Models, Husbandry, and Ethics

All animal experiments were performed in line with the directive 2010/63/EU and in accordance with the Royal Decree of 29 May 2013, found in the Belgian law. Approval was granted by the Ethical Commission for Animal Experimentation of the Vrije Universiteit Brussel (project 24-272-31). Female athymic nude mice (strain: Crl:NU(NCr)-Foxn1n^NU^; 5–10 weeks old, Charles River, L’Abresle, France) and C57BL/6J (strain: C57BL/6J; 5–10 weeks old, Charles River) were housed in individually ventilated cages (4 mice/cage) at 19–24 °C and a humidity of 40–60% with a 12 h/12 h day/night cycle. Water and food (SAFE^®^ 105; SAFE^®^ Complete Care Competence, Rosenberg, Germany) was provided ad libitum. Generally, mice were evaluated daily by visual inspection and monitored by weighing them every 2–3 days. Experiments were initiated after a one-week acclimatization period in our animal facility. Humane endpoints were met when general abnormal behaviour, abnormal posture, or prolapse was observed, and a weight loss of more than 20% as compared to the moment of the start of an experiment was reached. Follow-up of the mice and humane endpoints specific to an experiment are mentioned in the dedicated sections below. Sample sizes for each in vivo experiment were determined by G-Power analysis. An effect size d of 3.0 and a confidence interval of 95% were used.

### 2.6. In Vivo Biodistribution of sdAb 2BD42-s775z

Athymic nude Crl:NU(NCr)-Foxn1n^NU^ mice were subcutaneously (s.c.) inoculated on the top of the right hind limb with 10,000,000 SKOV-3 cells in a volume of 200 µL 1x PBS. Once the tumours reached a size ranging between 80 and 210 mm^3^, mice were randomly assigned to the experimental groups so that the mean tumour volume was similar over the experimental groups. Subsequently, mice were intravenously (i.v.) injected with 2 nmol, based on the concentration of the dye in a total volume of 150 µL 1x PBS, of sdAb 2BD42-s775z (*n* = 4) or the irrelevant control sdAb R3B23-s775z (*n* = 4). Subsequently, dorsal and ventral white light and fluorescence imaging was performed in the dark 1 h post-injection using a NIR fluorescence camera Fluobeam^®^ v1.47 (Fluoptics, Grenoble, France). Next, mice were killed, and organs of interest were collected to perform ex vivo white light and fluorescence imaging. Subsequently, fluorescence imaging of sdAb 2BD42-s775z was also evaluated longitudinally (*n* = 4) and was compared to OTL-38 (dose: 10 nmol; *n* = 4). Here, imaging was performed at 15 min, 30 min, 45 min, 1 h, 2 h, 4 h, 6 h, 8 h, 12 h, 16 h, 24 h, 48 h, 72 h, 96 h, 120 h, 144 h and 168 h post-injection. White light imaging was performed at an exposure time of 1000 ms, and fluorescence imaging was performed at exposure times of 10, 20, 30, 50, 75, 100, 150, 200, 300 and 500 ms. The imaging distance was set at 22.5 cm. Analysis was performed on images acquired at an exposure time of 150 ms for sdAb 2BD42-s775z and sdAb R3B23-s775z, and 30 ms for OTL-38 using ImageJ software version 1.54p (NIH, Bethesda, MD, USA). Regions of interest were drawn based on the white light images.

### 2.7. Validation of sdAb 2BD42-s775z in Localised and Metastatic Tumour Models

Athymic nude Crl:NU(NCr)-Foxn1n^NU^ mice were intraperitoneally (i.p.) inoculated with 10,000,000 SKOV-3 FLuc^+^ cells in a volume of 200 µL 1x PBS. From day 7 post-inoculation, follow-up of the tumour growth was performed weekly. D-Luciferin (150 mg/kg; PROMEGA, Madison, WI, USA) was i.p. injected to perform bioluminescence imaging (BLI) (PhotonIMAGER OPTIMA V4, Biospacelab, Nesles-La-Vallée, France) 10 min post-injection. Tumours were allowed to grow for a period of 4–6 weeks. Subsequently, mice were randomly divided into 2 groups and i.v. injected with 2 nmol of either sdAb 2BD42-s775z (*n* = 3) or the control sdAb R3B23-s775z (*n* = 3), based on the concentration of the dye in a total volume of 150 µL 1x PBS. Then, 50 min post-injection of the tracers, D-Luciferin (150 mg/kg; PROMEGA, USA) was i.p. injected, and 5 min later, mice were killed via Dolethal^®^ injection (300 µL at a concentration of 200 mg/mL, 1/3 diluted in 0.9% NaCl; Vétoquinol, France). Subsequently, the peritoneal cavity was opened. Kidneys were removed, and the bladder was covered by a black paper so that tumour lesions located near these organs would not be missed.

Next, white light imaging and fluorescence imaging (Fluobeam^®^; Fluoptics) were performed before resection to confirm the presence of multiple small tumour lesions. Finally, tumour lesions were removed based on the fluorescent signal. Fluorescence imaging and analysis was performed as described above. Analysis of the BLI images was performed in M3Vision software (Biospacelab). Specific humane endpoints for this experiment were severe complications such as peritonitis, laceration of abdominal organs or (paralytic) ileus, and the presence of ascites.

In a final in vivo experiment, C57BL/6J mice were intrapulmonary inoculated with 100,000 TC-1 hFRα^+^ FLuc^+^ cells in a volume of 10 µL 1x PBS into the 4th and the 5th intercostal space at a depth of 4–5 mm. From day 6 post-inoculation, tumour growth follow-up was performed twice a week by both µCT imaging (MOLECUBES, Ghent, Belgium) and BLI. Then, 1–2 weeks post-inoculation, mice were randomly divided into 2 groups and i.v. injected with 2 nmol of sdAb 2BD42-s775z (*n* = 4) or the control sdAb R3B23-s775z (*n* = 4), based on the concentration of the dye in a total volume of 150 µL 1x PBS. After 50 min post-injection of the different tracers, D-Luciferin (150 mg/kg; PROMEGA, USA) was i.p. injected. Subsequently, 1 h post-injection of the tracers, mice were killed, and the thoracic cavity was opened to perform in situ and ex vivo white light imaging, BLI (only ex vivo), and fluorescence imaging of the lungs. Imaging and analysis were performed as described above. Humane endpoints were mainly related to the intrapulmonary injections such as paralysis of the hind legs due to injection into the spine and laboured respiration.

### 2.8. Statistics

Statistical analyses were performed using GraphPad Prism v10.4.1. Normality of the data was assessed using the Shapiro–Wilk test. An unpaired one-tailed *t*-test was used to compare the mean fluorescent intensity of the fluorescently labelled sdAb 2BD42 with the control condition for the in vitro cell binding study. The uptake of the different compounds in the tumour and organs of interest was analysed using the Mann–Whitney test. Subsequent analysis of the different tumour-to-background ratios was performed using an unpaired one-tailed *t*-test with Welch’s correction. Statistical significance was set at *p* < 0.05 (* *p* < 0.05, ** *p* < 0.01, *** *p* < 0.001, **** *p* < 0.0001). Descriptive statistics are represented as mean ± standard deviation (SD).

## 3. Results

### 3.1. Fluorescence Labelling of sdAb 2BD42 and Quality Control

**Figure 1:**
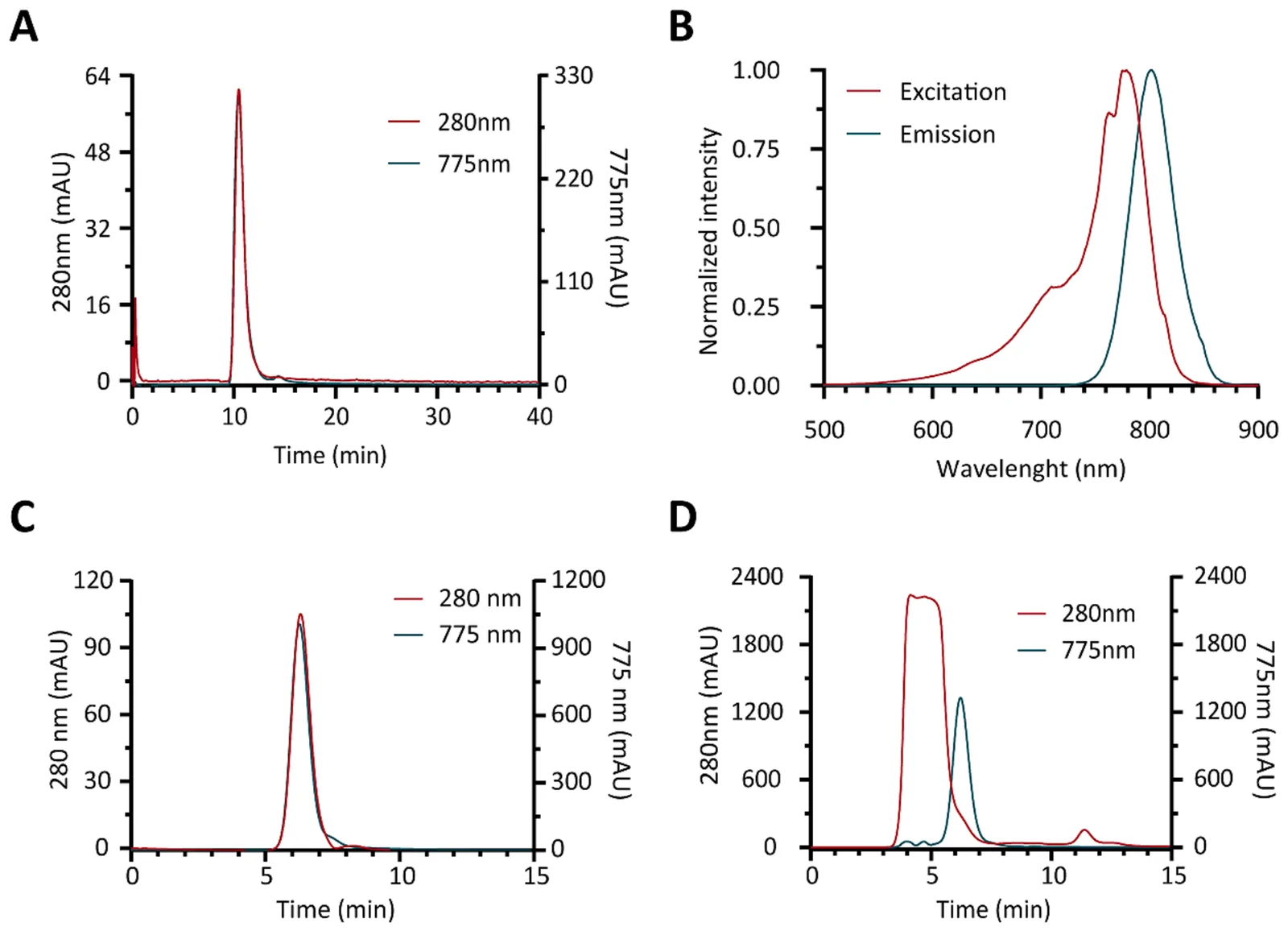
Fluorescence labelling of sdAb 2BD42 and quality control. Quality control of (**A**) sdAb 2BD42-s775z performed by analytical size-exclusion chromatography indicating a pure product. (**B**) Excitation and emission spectra of sdAb 2BD42-s775z. (**C**) Stability of sdAb 2BD42-s775z at 24 h at 21 °C and (**D**) at 1 h in the presence of human serum at 37 °C.

The anti-hFRα sdAb 2BD42 and the negative control sdAb R3B23 were randomly labelled with the dye s775z via NHS-chemistry. Purity of both tracers was confirmed via analytical SEC (Figure 1A, Supplementary Figure S1A). A mixture of different DOLs were observed by mass spectrometry and SDS-PAGE and resulted in an average DOL of 1.3 and 1.1, respectively (Supplementary Figure S1B,C). Furthermore, spectral characteristics of sdAb 2BD42-s775z (ex/em maxima: 779/802 nm) remained close to those of the free dye (ex/em maxima: 775/794 nm) (Figure 1B). Stability above 95% was demonstrated up to 24 h in 1x PBS at 21 °C, and no binding to human serum was observed. In human serum, the tracer also remained stable for 1 h post-incubation at 37 °C (Figure 1C,D).

### 3.2. In Vitro Functionality Assessment of sdAb 2BD42-s775z

**Figure 2:**
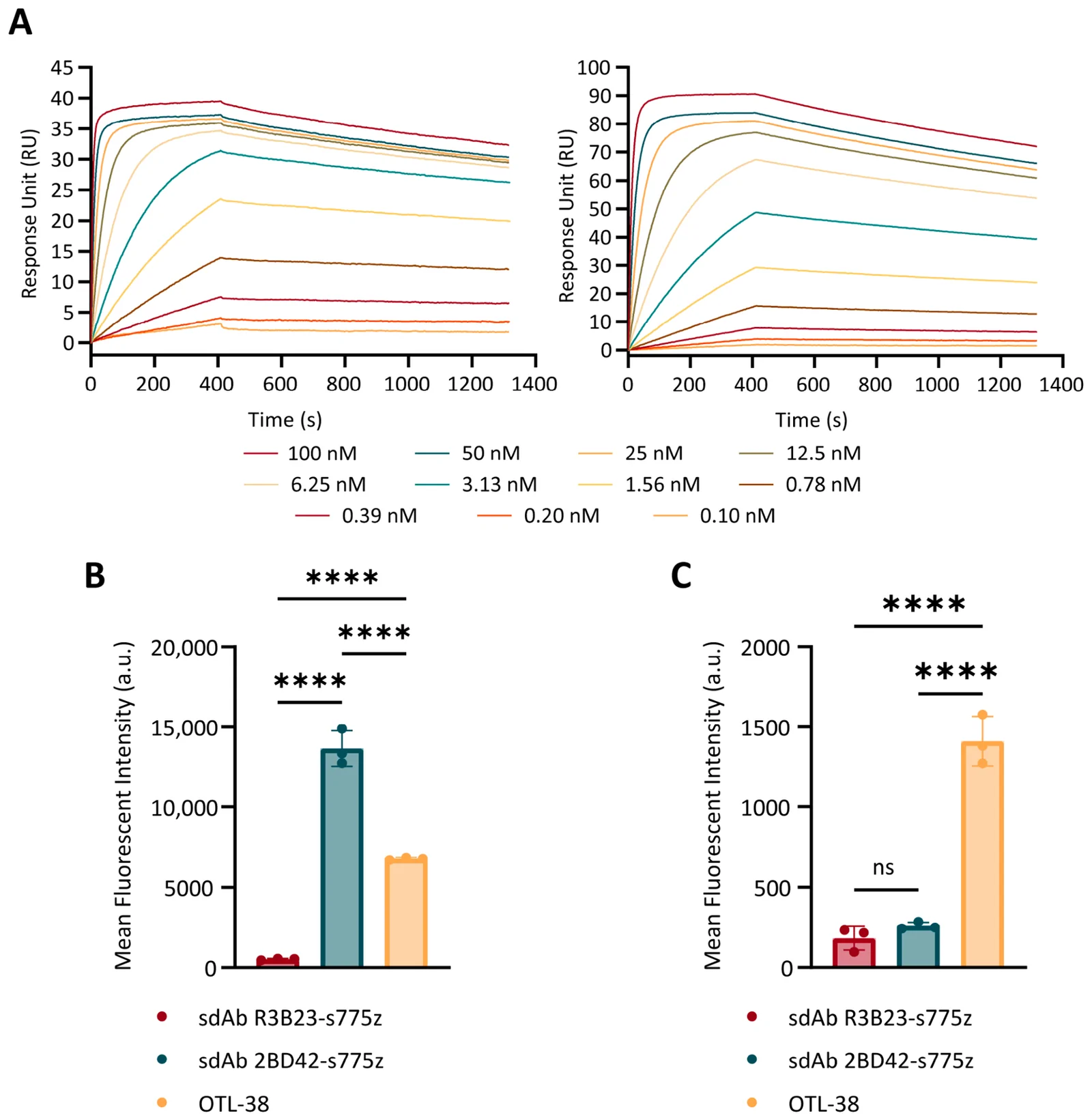
In vitro functionality assessment of sdAb 2BD42-s775z. (**A**) Sensograms of different concentrations (as indicated in the graph) of sdAb 2BD42 and sdAb 2BD42-s775z on hFRα measured by SPR. Kinetics were measured with a two-fold dilution series of the sdAbs (100 nM–0.10 nM). Fitting of the curves was performed using a 1:1 binding model. Cell binding study of 100 nM sdAb 2BD42-s775z and OTL-38 on (**B**) hFRα-expressing SKOV-3 cells and on (**C**) hFRβ-expressing THP-1 cells. Experiments were performed in triplicate (*n* = 3). Error bars on the graph represent the standard deviation. ns = not significant; statistical significance was set at *p* < 0.05 (**** *p* < 0.0001).

First, the affinity and kinetic properties of sdAb 2BD42-s775z to hFRα were assessed via SPR. Here, the sub-nanomolar affinity of the tracer for the receptor was maintained after conjugation of the dye (K_D 2BD42_ = 0.13 ± 0.01 nM; K_D 2BD42-s775z_ = 0.44 ± 0.03 nM) (Figure 2A). Association and dissociation rates can be found in Supplementary Table S1. Additionally, a cell binding study on hFRα-expressing SKOV-3 cells was performed to assess the specificity of sdAb 2BD42-s775z, and the cells were compared to OTL-38 and the irrelevant control sdAb R3B23-s775z. Both sdAb 2BD42-s775z and OTL-38 showed specific binding to SKOV-3 cells compared to sdAb R3B23-s775z (Figure 2B). As binding of OTL-38 to hFRβ-expressing macrophages has been described in the literature, we wanted to confirm that our tracer was able to discriminate between hFRα and hFRβ. Therefore, a cell binding study on hFRβ-expressing THP-1 monocytes was performed. As can be seen in Figure 2C, OTL-38 showed significantly higher binding on THP-1 cells, while no binding of sdAb 2BD42-s775z could be observed (Figure 2C).

### 3.3. In Vivo Biodistribution of sdAb 2BD42-s775z in a Subcutaneous Ovarian Cancer Tumour Model

**Figure 3:**
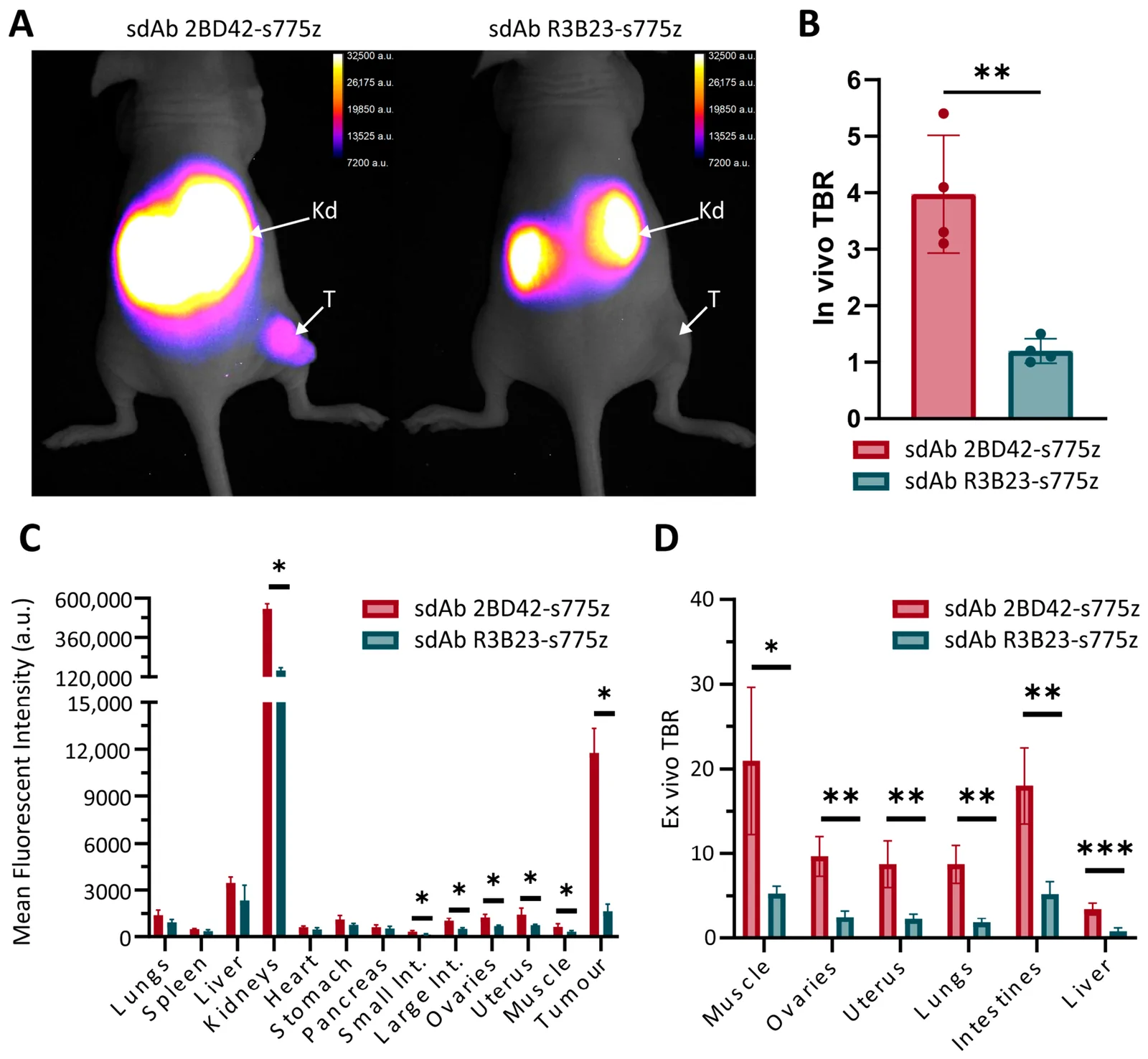
Fluorescence imaging of sdAb 2BD42-s775z and the irrelevant control Nb R3B23-s775z in a subcutaneous SKOV-3 tumour model. (**A**) Dorsal fluorescent images 1 h post-injection of sdAb 2BD42-s775z (left; *n* = 4) and the irrelevant control sdAb R3B23-s775z (right; *n* = 4). The kidneys (Kd) and tumour (T) are indicated by arrows. Scale bars on the images show the minimum and maximum fluorescent intensity in arbitrary units (a.u.). The images shown in this figure depict the most representative images of the study. (**B**) In vivo TBR of sdAb 2BD42-s775z and the control sdAb R3B23-s775z at 1 h post-injection. (**C**) Mean fluorescent intensity in dissected organs of sdAb 2BD42-s775z and sdAb R3B23-s775z 1 h post-injection of the tracers. (**D**) Ex vivo TBR in relevant organs of sdAb 2BD42-s775z and sdAb R3B23-s775z at 1 h post-injection. Statistical analysis was performed using the Mann–Whitney test. Statistical significance was set at *p* < 0.05. Error bars on the graphs represent the standard deviation. Statistical significance was set at *p* < 0.05 (* *p* < 0.05, ** *p* < 0.01, *** *p* < 0.001).

Fluorescence dorsal and ventral imaging of sdAb 2BD42-s775z was performed in nude mice bearing s.c. SKOV-3 tumours. For sdAb 2BD42-s775z, dorsal imaging revealed an in vivo TBR of around 4 at 1 h post-injection, which was significantly higher than the in vivo TBR around 1 of sdAb R3B23-s775z (Figure 3A,B). Following dissection of the mice at 1 h post-injection, ex vivo fluorescence imaging of the organs of interest confirmed high uptake of the targeting tracer in the tumour (11,766 ± 1447 a.u.) compared to when mice were injected with the irrelevant control sdAb R3B23-s775z (1671 ± 470 a.u.; *p* = 0.01). Furthermore, low to no signal was observed in other healthy organs using sdAb 2BD42-s775z except for high signal in the kidneys (537,923 ± 33,558 a.u.; renal clearance) and slightly elevated signals in the liver (3588 ± 434 a.u.) (Figure 3C; Supplementary Figure S2). Ex vivo TBRs were calculated for muscle (21.0 ± 9.1), ovaries (10.1 ± 2.4), uterus (8.8 ± 2.8), lungs (8.7 ± 2.3), intestines (18.0 ± 4.5), and liver (3.5 ± 0.7) and were high enough to be considered relevant for FGS and statistically significantly higher than the control (Figure 3D).

**Figure 4:**
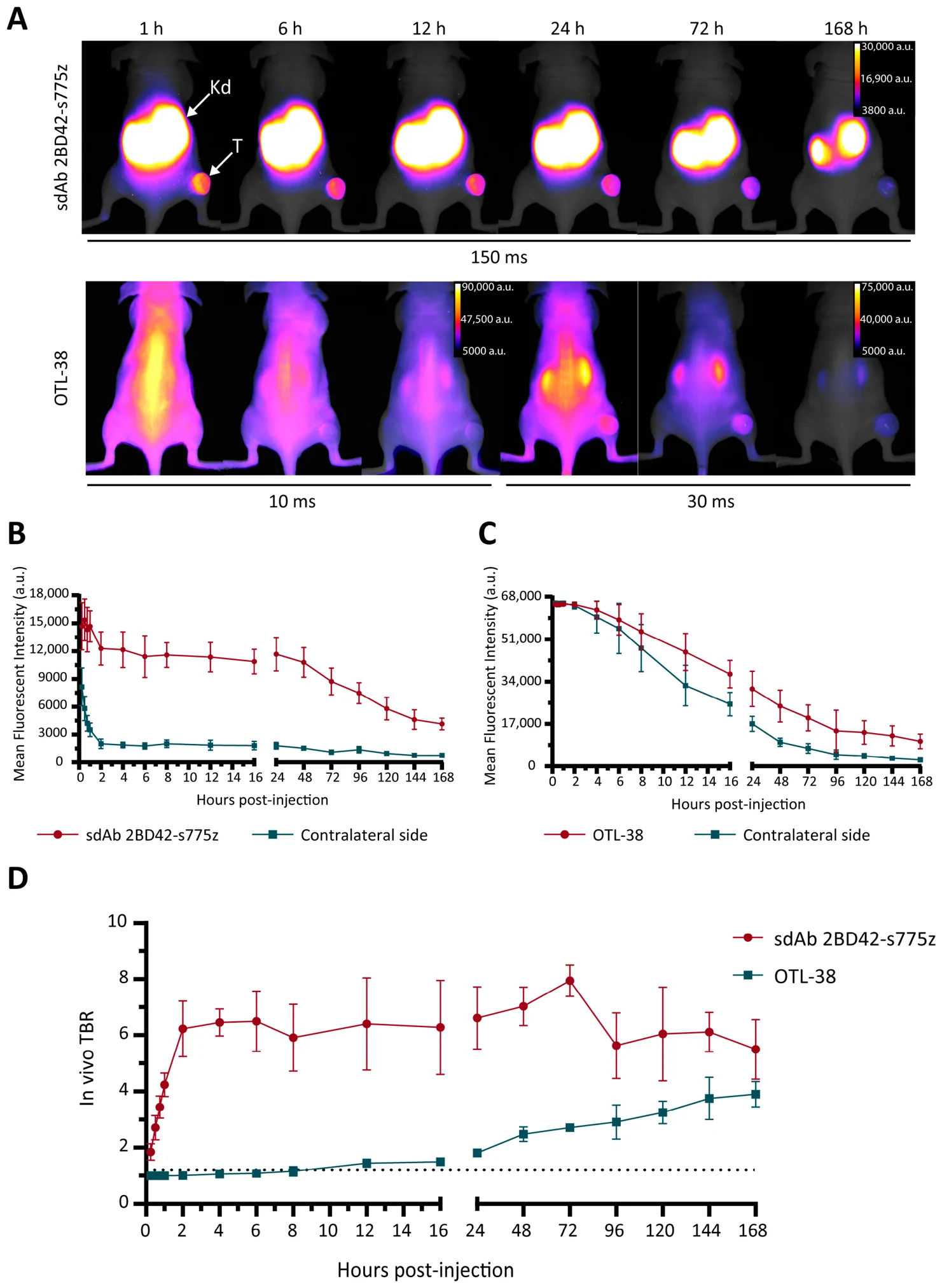
Fluorescence longitudinal imaging of sdAb 2BD42-s775z and OTL-38 in a subcutaneous SKOV-3 tumour model. (**A**) In vivo dorsal fluorescence imaging of mice bearing subcutaneous SKOV-3 tumours injected with either sdAb 2BD42-s775z (*n* = 4) or OTL-38 (*n* = 4) at 1 h, 6 h, 12 h, 24 h, 72 h and 168 h post-injection. Images shown for sdAb 2BD42-s775z were acquired at an exposure time of 150 ms. Images shown for OTL-38 were acquired at an exposure time of 10 ms for 1 h, 6 h and 12 h post-injection and at an exposure time of 30 ms for 24 h, 72 h and 168 h post-injection. Quantification of the signal was performed on images acquired at an exposure time of 150 ms and 30 ms for sdAb 2BD42-s775z and OTL-38, respectively. Scale bars on the images show the minimum and maximum fluorescent intensity in arbitrary units (a.u.). The images shown in this figure depict the most representative images of the study. Mean fluorescent intensity (MFI) of (**B**) sdAb 2BD42-s775z and (**C**) OTL-38 measured by in vivo fluorescence dorsal imaging over time up to 168 h post-injection. The MFI of each tracer was compared to the MFI of the background signal measured at the contralateral side of the tumour. (**D**) In vivo TBRs of sdAb 2BD42-s775z and OTL-38 determined by longitudinal fluorescence dorsal imaging up to 168 h post-injection. The in vivo TBR of sdAb R3B23-s775z is indicated as a dotted line on the graph. Error bars on the graphs represent the standard deviation.

Longitudinal fluorescence dorsal imaging following administration of sdAb 2BD42-s775z further showed that the high signal in the tumour was retained up to 1 week post-injection, while background signal intensity decreased rapidly (Figure 4A,B). This resulted in an in vivo TBR of 4.1 ± 0.9 as soon as 1 h post-injection, with a maximum TBR of 8.8 ± 1.4 at 72 h post-injection, after which it slowly started to decrease until 5.3 ± 1.0 at 168 h post-injection (Figure 4D). In contrast, longitudinal imaging of OTL-38 resulted in a more gradual decline in the background signal intensity, which closely paralleled the intensity in the tumour (Figure 4A,C). The highest in vivo TBR for OTL-38 of 4.2 ± 0.4 was reached at 168 h post-injection (Figure 4D).

### 3.4. Validation of sdAb 2BD42-s775z in Localised and Metastatic Tumour Models

**Figure 5:**
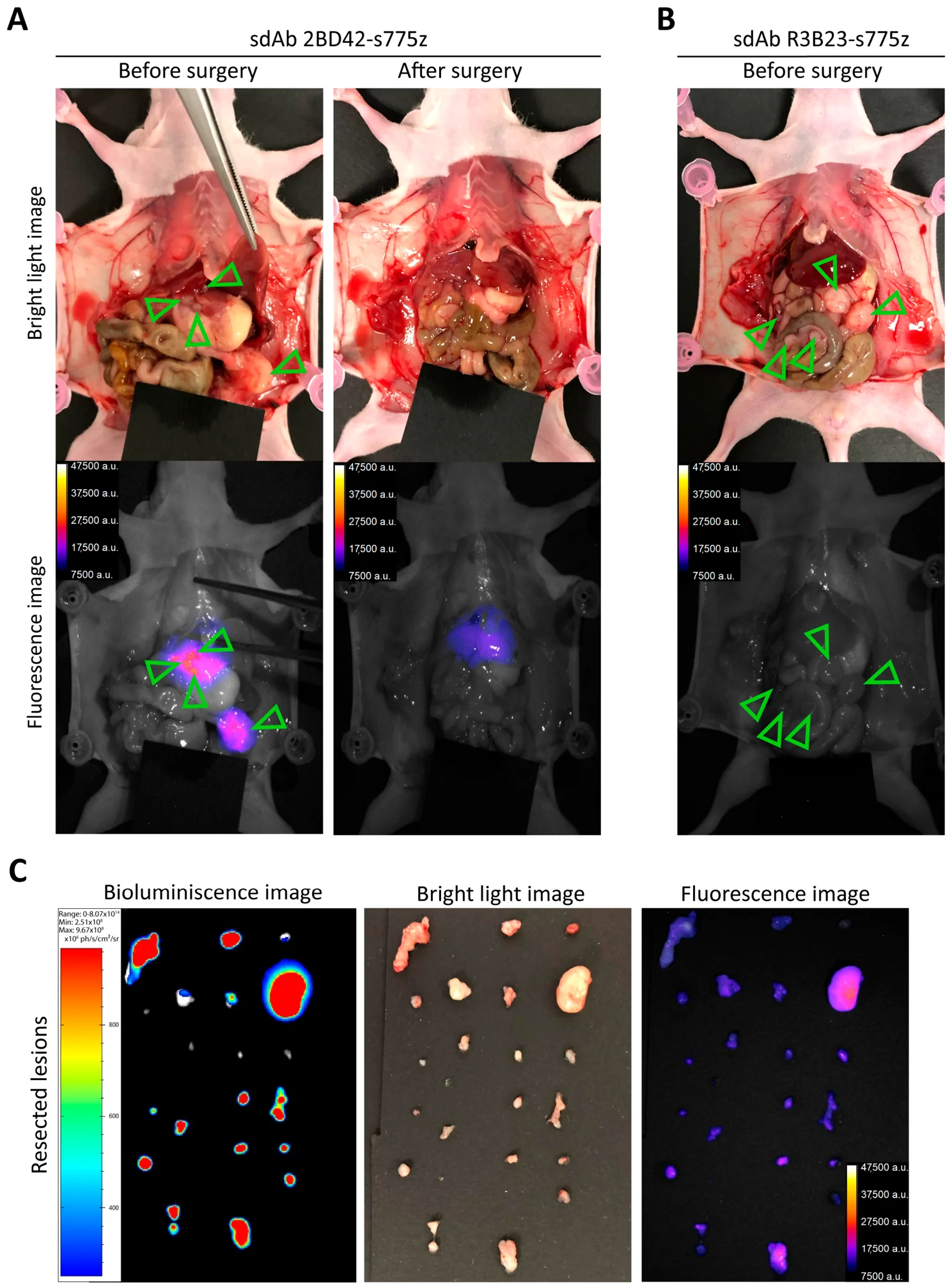
sdAb 2BD42-s775z in an ovarian carcinomatosis tumour model. (**A**) Bright light (**top**) and fluorescence imaging (**bottom**) of intraperitoneal tumour lesions 1 h post-injection of sdAb 2BD42-s775z before (**left**) and after (**right**) surgery (*n* = 3). (**B**) Bright light (**top**) and fluorescence imaging (**bottom**) of intraperitoneal tumour lesions 1 h post-injection of the irrelevant control sdAb R3B23-s775z before surgery (*n* = 3). Intraperitoneal lesions in situ are indicated by green arrows (Δ). (**C**) BLI (**left**), bright light (**middle**) and fluorescence imaging (**right**) of the resected lesions after surgery 1 h post-injection of sdAb 2BD42-s775z. Scale bars on the fluorescent and BLI images show the minimum and maximum intensity in arbitrary units (a.u.) and ph/s/cm^2^/sr, respectively. The images shown in this figure depict the most representative images of the study.

As ovarian cancer is most often diagnosed in stage 3–4, in which cancer lesions have spread into the abdominal cavity, we evaluated if the tracer was able to highlight small tumour lesions in an ovarian i.p. carcinomatosis tumour model. Nude mice bearing i.p. tumour lesions were intravenously injected with either the fluorescently labelled targeting sdAb or the control sdAb. Subsequently, mice were killed, the peritoneal cavity was opened, and the kidneys were removed to perform bright light and fluorescence imaging 1 h post-injection of the tracers. Upon opening of the abdominal cavity, multiple tumour lesions could be observed using bright light and fluorescence imaging using sdAb 2BD42-s775z (Figure 5A). Subsequently, highlighted tumour lesions were resected solely based on the fluorescent signal. Once the surgery was complete, no residual tumour lesions in the peritoneal cavity could be detected using bright light or fluorescence imaging (Figure 5A). Finally, the resected lesions highlighted by fluorescence were evaluated using BLI. Here, it was confirmed that all resected lesions were in fact tumour lesions (Figure 5C). In contrast, when the irrelevant control sdAb R3B23-s775z was injected, no tumour lesions could be detected using fluorescence imaging while their presence was confirmed by bright light imaging (Figure 5B). Consequently, no lesions were resected based on the fluorescent signal, which resulted in the presence of residual tumour tissue post-surgery, which was confirmed by bright light imaging (Supplementary Figure S3).

**Figure 6:**
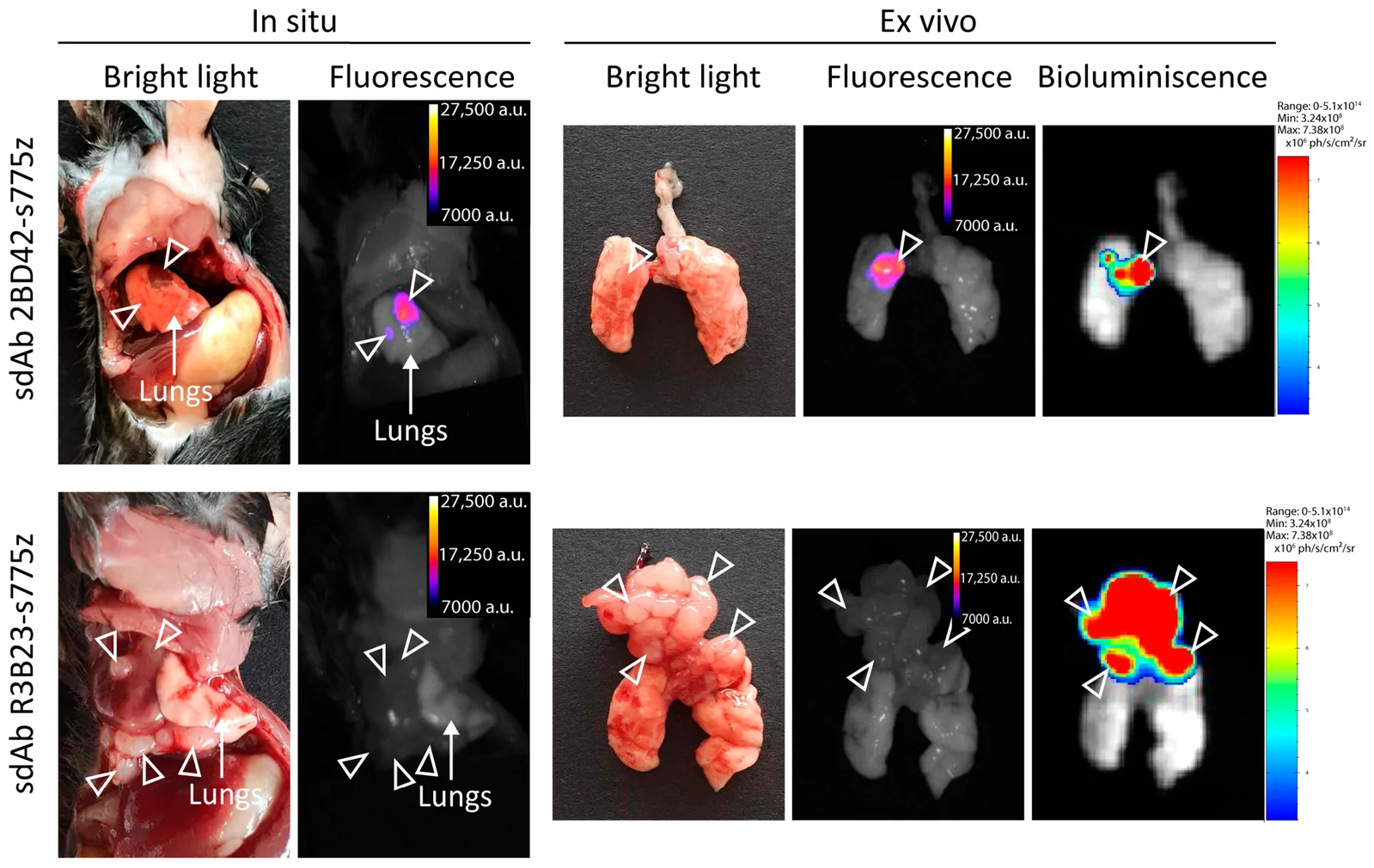
Evaluation of sdAb 2BD42-s775z in an orthotopic lung tumour model. In situ (**left**) bright light and fluorescence imaging and ex vivo (**right**) bright light imaging, fluorescence imaging, and BLI of mice bearing orthotopic TC-1 hFRα+ FLuc+ lung tumours 1 h post-injection of sdAb 2BD42-s775z (top; *n* = 4) or the irrelevant control sdAb R3B23-s775z (bottom; *n* = 4). Tumours are indicated with white Δ on all images. Scale bars on the fluorescent and BLI images show the minimum and maximum intensity in arbitrary units (a.u.) and ph/s/cm^2^/sr, respectively. The images shown in this figure depict the most representative images of the study.

As an additional PoC for the use of sdAb 2BD42-s775z as a tracer for FGS in multiple tumour types, intraoperative imaging was mimicked in an orthotopic lung tumour model. Tumour growth follow-up was initially performed via µCT imaging and BLI (Supplementary Figure S4A,B). Following exposure of the thoracic cavity, lungs were imaged using bright light and fluorescence imaging 1 h post-injection of the tracer. Fluorescent signals could clearly be observed in multiple lung tumour lesions using sdAb 2BD42-s775z both in situ and ex vivo. Contrarily, and similar to the ovarian cancer model, no lung tumours could be detected by fluorescence imaging in mice injected with the irrelevant control sdAb R3B23-s775z in situ and ex vivo, while the presence of tumour lesions was confirmed by bright light imaging and BLI (Figure 6).

## 4. Discussion

The clinical adoption of molecular FGS is accelerating, exemplified by the approval and clinical adoption of Cytalux^™^ for ovarian and lung cancer surgery. However, improving tumour specificity remains an important area of ongoing research. In the present study, we aimed to preclinically validate 2BD42-s775z, a novel sdAb-based targeted tracer that selectively targets the alpha isoform of hFR, for intraoperative tumour detection. In addition, this work also expands the applicability of sdAb 2BD42, as it has already been preclinically validated for PET imaging and radioligand therapy [27,28].

To do so, the sdAb was fluorescently labelled to a NIR cyanine-based fluorophore. As has already been described, the choice of fluorophore may significantly influence the tracer’s pharmacokinetic behaviour in vivo [34]. Therefore, we opted for the dye s775z as this dye has improved stability and hydrophilicity and is less prone to photobleaching compared to other NIR dyes emitting light around 800 nm [35]. These characteristics ensure that the favourable biodistribution profile of sdAbs is retained [30,36]. This was confirmed in our study, as high uptake in the tumour was observed as soon as 1 h post-injection with low background signals in healthy organs. As expected, prominent fluorescent signals were also observed in the kidneys and the bladder due to the predominantly renal clearance of sdAbs. In a clinical setting, however, the high renal signal is expected to be less of an issue because of the greater anatomical distances between organs and the relatively limited field of view of laparoscopic systems [37]. Furthermore, the renal fascia is likely to substantially attenuate fluorescence originating from the kidneys. Although some hepatic uptake was observed, the liver signal remained relatively low, particularly compared with sdAbs labelled with IRDye800CW [30]. This benefits the detection and resection of tumour lesions located on or near the liver.

The fluorescent intensity of sdAb 2BD42-s775z and OTL-38 could not be compared directly, as a dose of 2 nmol was used for sdAb 2BD42-s775z, while a dose of 10 nmol was used for OTL-38, which is the most commonly used dose in preclinical studies [38,39]. This resulted in different acquisition times for the analysis. In addition, both tracers contain a different fluorophore, which further complicates the quantification of the fluorescent signal because of differences in their spectral compatibility with the camera’s detection characteristics [40]. Therefore, TBR was chosen as more robust metric to compare both tracers, as it inherently accounts for blood clearance, tissue extravasation, and penetration, independently of the absolute signal intensity difference arising from dose or fluorophore. It should, however, be noted that TBRs can also be influenced by the administered dose and thus should be interpreted with caution. Using sdAb 2BD42-s775z, a significant TBR of around 3 was achieved as early as 1 h post-injection, while OTL-38 required a minimum of 24 h post-injection to reach a clinically relevant TBR of 1.5 [41]. A delayed optimal TBR has already been reported by Predina et al., who described a TBR of approximately 3 at 24 h post-injection of OTL-38 in a preclinical lung tumour model [42]. Similar TBRs for Cytalux™ were observed in a clinical study of patients with lung adenocarcinomas [43]. Furthermore, it has been reported that the optimal imaging timepoint of Cytalux™ is 1–9 h post-injection for ovarian cancer and 1–24 h post-injection for lung cancer [44]. However, long injection-to-imaging time intervals are not preferable and could be omitted by the use of sdAb 2BD42-s775z.

While sdAbs are known to provide high-contrast tumour imaging shortly after administration, the tumour signal often decreases rapidly over time. Interestingly, sdAb 2BD42-s775z exhibited sustained tumour retention, resulting in high tumour-associated fluorescence and relevant TBRs for up to one week post-injection. This sustained signal is most likely attributable to the tracer’s very slow dissociation rate, yielding a sub-nanomolar affinity. This may be advantageous during lengthy or delayed surgical procedures while preserving the practical logistical benefit of the tracer administration shortly before surgery.

To evaluate the tracer under more clinically relevant conditions, we assessed its performance in an intraperitoneal ovarian carcinomatosis model, reflecting the advanced stage disease at which ovarian cancer is commonly diagnosed. In this setting, sdAb 2BD42-s775z enabled the clear visualisation of multiple tumour lesions throughout the peritoneal cavity. Moreover, the tracer successfully identified localised lung tumours, allowing clear discrimination between malignant and healthy lung tissue. In both models, no tumour-specific signal was observed with the irrelevant control sdAb R3B23-s775z, underlining the high specificity of sdAb 2BD42-s775z.

However, as the anti-hFRα sdAb 2BD42 is not cross-reactive with murine FRα and the evaluation of the biodistribution profile was performed in a xenograft mouse model, the low background signal observed in normal organs in this study may not fully reflect ‘on-target, off-tumour uptake’ in healthy human FRα-expressing tissues. Biodistribution studies with the radiolabelled analogue [^99m^Tc]Tc-sdAb 2BD42 in naive hFRα^+/+^ knock-in mice demonstrated significantly higher tracer accumulation in the choroid plexus and ovaries, both tissues known to express FRα, whereas no appreciable uptake was detected in other organs by SPECT/CT imaging. Furthermore, immunohistochemical analysis revealed only low-level hFRα expression in the kidney tubules, splenic cells, and pancreatic acinar cells [28]. These findings suggest that physiological hFRα expression is relatively restricted and that off-tumour uptake of sdAb 2BD42 is likely to be limited. While the exact impact on TBR in humans remains to be established, the biodistribution profile of the radiolabelled analogue supports the expectation that clinically relevant TBRs will be achieved in patients.

The additional binding of Cytalux™ to hFRβ, which is expressed, for instance, on activated macrophages residing in lymph node sinuses, can complicate the identification of lymph node metastasis by generating fluorescence in non-malignant lymphatic tissue [17]. In this study, we demonstrated that sdAb 2BD42-s775z does not bind to hFRβ-expressing cells. This was expected, as no binding to hFRβ or hFRϒ was observed by ELISA during the generation and screening of sdAb 2BD42 [28]. Therefore, the selective recognition of hFRα may improve tumour specificity and reduce on-target off-tumour fluorescence arising from hFRβ-expressing immune cells. On the other hand, binding of Cytalux™ to hFRβ-expressing tumour-associated macrophages within the tumour microenvironment may enhance the overall fluorescent signal, as both the tumour cells and the stromal compartment contribute to tracer accumulation and signal intensity [45]. We do have to acknowledge that in this study, it was not possible to assess the potential advantage of avoiding hFRβ binding for the detection of lymph node metastasis, as this would require a humanised mouse model expressing both hFRα and hFRβ. Therefore, further studies are needed to evaluate the relative benefits of selective hFRα targeting versus dual hFRα/hFRβ recognition.

## 5. Conclusions

To conclude, this study describes the preclinical validation of the anti-hFRα sdAb 2BD42-s775z for FGS, using ovarian and lung cancer as representative tumour models. The tracer demonstrated high purity and stability, selectively bound hFRα-expressing cells without cross-reacting with hFRβ-expressing cells, and demonstrated favourable biodistribution with high tumour uptake and low background fluorescence. Consequently, clinically relevant TBRs were achieved as early as 1 h post-injection and maintained for up to one week. Moreover, sdAb 2BD42-s775z demonstrated its relevance in an ovarian carcinomatosis tumour model and an orthotopic lung tumour model. These findings support the potential of sdAb 2BD42-s775z as a highly specific tracer for intraoperative tumour detection. Whether selective hFRα targeting offers an advantage over currently available folate receptor-targeted tracers for the detection of lymph node metastasis remains to be established in appropriate humanised in vivo models.

## Data Availability

The datasets generated and/or analysed during this study are available from the corresponding author on reasonable request.

## Supplementary Materials

The following supporting information can be downloaded at https://www.mdpi.com/article/10.3390/biom16101426/s1, Figure S1: Fluorescence labelling of sdAb R3B23-s775z and QC of sdAb 2BD42-s775z; Figure S2: Ex vivo fluorescence imaging of sdAb 2BD42-s775z and sdAb R3B23-s775z in a subcutaneous SKOV-3 tumour model; Figure S3: sdAb R3B23-s775z in an ovarian carcinomatosis tumour model after surgery; Figure S4: Tumour growth follow-up of orthotopic lung tumours; Table S1: Affinity determination of sdAb 2BD42-s775z by Surface Plasmon Resonance.

## Abbreviations

The following abbreviations are used in this manuscript:

BLI	Bioluminescence imaging
DOL	Degree of labelling
FGS	Fluorescence-guided surgery
FLuc	Firefly luciferase
HBS	HEPES-buffered saline
hFR	Human folate receptor
hFRα	Human folate receptor alpha
hFRβ	Human folate receptor beta
i.p.	Intraperitoneal
i.v.	Intravenous
k_a_	Association rate constant
k_d_	Dissociation rate constant
K_D_	Equilibrium dissociation constant
MFI	Mean fluorescent intensity
NHS	N-hydroxysuccimide
NIR	Near-infrared
PBS	Phosphate-buffered saline
PoC	Proof of concept
s.c.	Subcutaneous
sdAb	Single-domain antibody
SEC	Size-exclusion chromatography
SPR	Surface plasmon resonance
TBR	Tumour-to-background ratio
