# Supplementary material for: Validation of a Highly Specific Anti-Human Folate Receptor Alpha Single-Domain Antibody for Rapid Fluorescence-Guided Surgery of Ovarian and Lung Cancer
Source: Biomolecules. 2026 Oct 1;16(10):1426. doi: 10.3390/biom16101426 (PMC13702722; doi:10.3390/biom16101426)
Supplement: Supplementary file 1 [file biomolecules-16-01426-s001.zip › biomolecules-4576224-supplementary.pdf]

Validation of a Highly Specific Anti-Human Folate Receptor Alpha Single-Domain Antibody for Rapid Fluorescence-Guided Surgery of Ovarian and Lung Cancer

Sam Floru [1], Stijn Vandorpe [1], Laurent Navarro [2], Shivakrishna Kallepu [3], Madduri Srinivasarao [3], Philip S Low [3], Matthias D'Huyvetter [1,2] and Sophie Hernot [1*]

[1] Vrije Universiteit Brussel (VUB), Brussels health Campus/Faculty of Medicine and Pharmacy, Molecular Imaging and Therapy Research group (MITH), Laarbeeklaan 103, 1090 Brussels, Belgium; Sam.Floru@vub.be; Stijn.vandorpe@gmail.com; Matthias.DHuyvetter@vub.be; Sophie.Hernot@vub.be

[2] Precirix NV, Brussels, Belgium; laurent.n@hotmail.com

[3] Institute for Drug Discovery, Purdue University, West Lafayette, IN, USA; shivakrishna.kallepu@mof-fitt.org; msriniva@purdue.edu; plow@purdue.edu

* Correspondence: Sophie.Hernot@vub.be

Supplementary figures

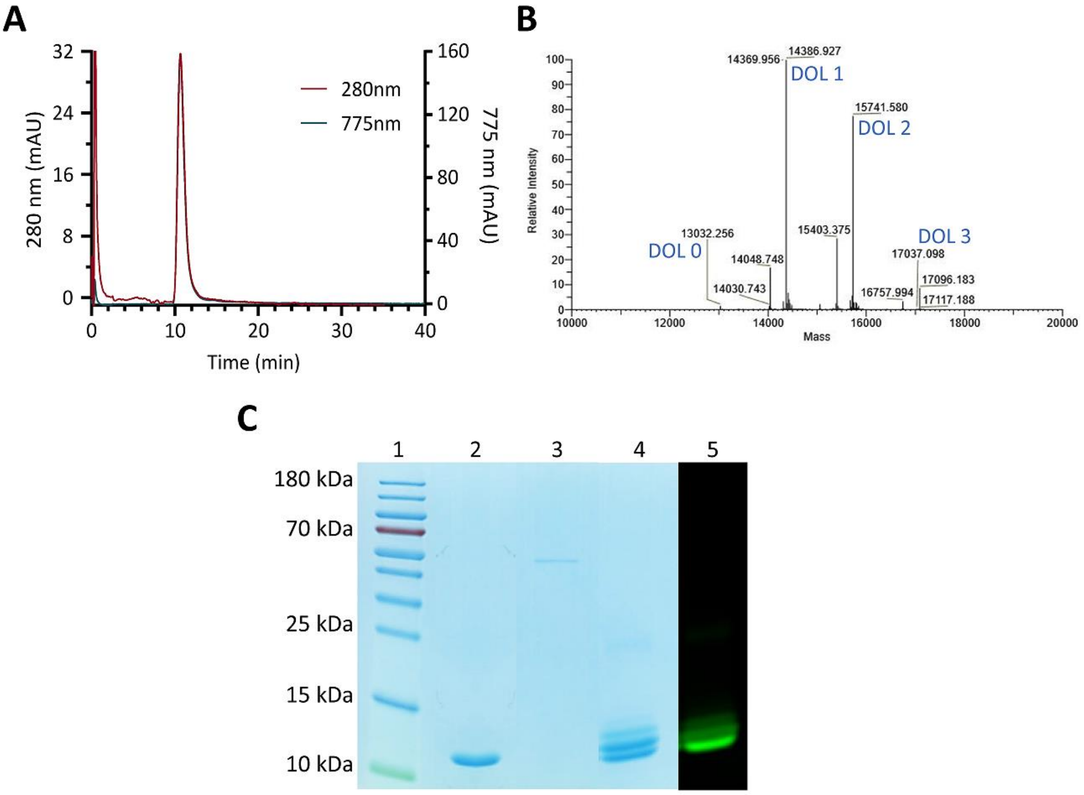

Figure S1. Fluorescence labeling of sdAb R3B23-s775z and QC of sdAb 2BD42-s775z. Quality control of (**A**) sdAb R3B23-s775z performed by analytical size exclusion chromatography indicating a pure product. (**B**) Mass spectrometry (ESI-Q-TOF-MS) of sdAb 2BD42-s775z showing a mixture of DOL 0,1,2 and 3 of the fluorescently labeled sdAb. (**C**) SDS-PAGE of unlabeled sdAb 2BD42 (band 2) and labeled sdAb 2BD42-s775z indicating the different degree of labeling of the product as shown by Instant Blue staining (band 4) and fluorescence imaging (band 5). Band 3 shows the positive control BSA and the protein ladder is shown in band 1.

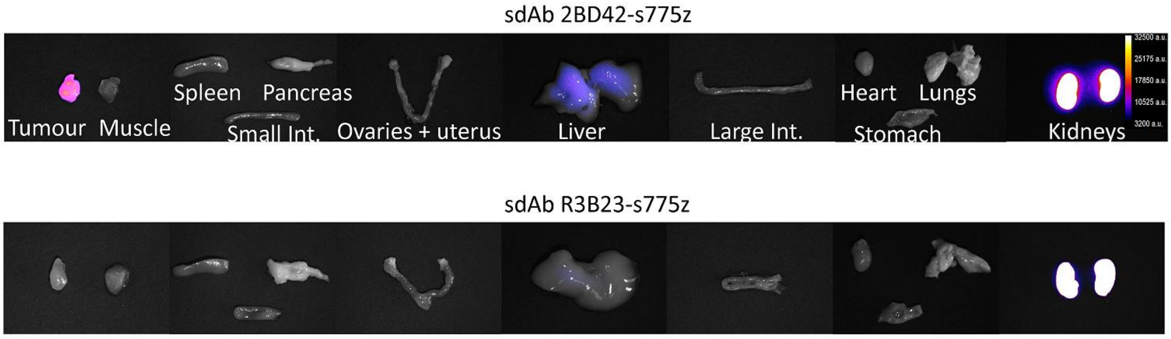

Figure S2. Ex vivo fluorescence imaging of sdAb 2BD42-s775z and sdAb R3B23-s775z in a subcutaneous SKOV-3 tumour model. Ex vivo fluorescence imaging of dissected organs 1 h post-injection of sdAb 2BD42-s775z (top) or the irrelevant control sdAb R3B23-s775z (bottom). Scale bars on the images show the minimum and maximum fluorescent intensity in arbitrary units (a.u.). The images shown in this figure depict the most representative images of the study.

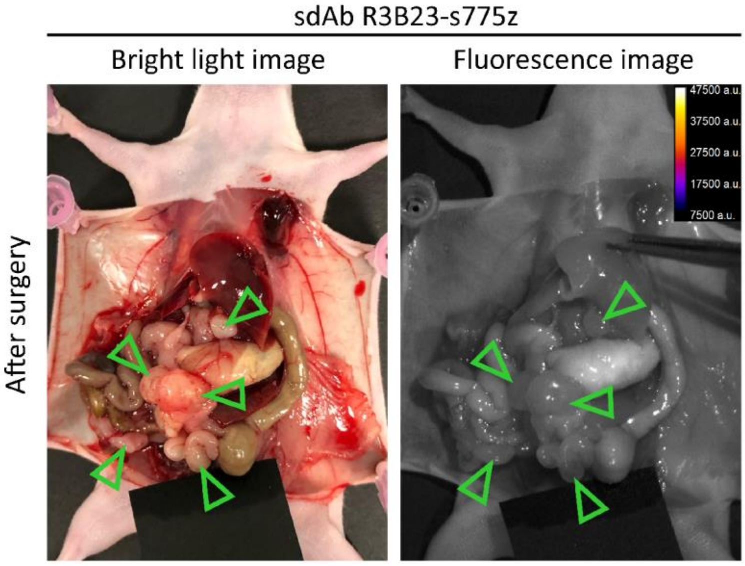

Figure S3. sdAb R3B23-s775z in an ovarian carcinomatosis tumour model after surgery. Bright light (left) and fluorescence imaging (right) of intraperitoneal tumour lesions 1 h post-injection of the irrelevant control sdAb R3B23-s775z after surgery. Intraperitoneal lesions *in situ* are indicated by green arrows (Δ). Scale bars on the images show the minimum and maximum fluorescent intensity in arbitrary units (a.u.). The images shown in this figure depict the most representative images of the study.

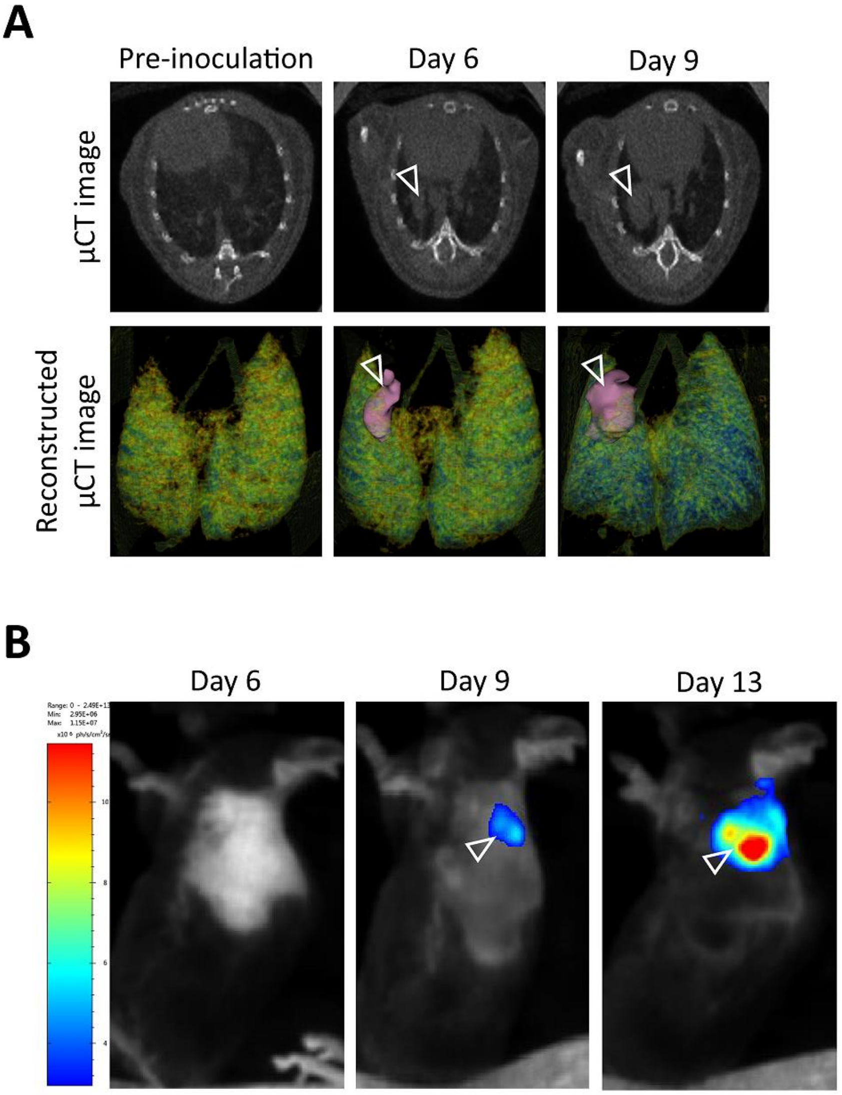

Figure S4. Tumour growth follow-up of orthotopic lung tumours. Orthotopic lung tumour growth follow-up by (**A**) μCT imaging at day 0, 6 and 9 post-inoculation and (**B**) BLI at day 6, 9 and 13 post-inoculation. Tumours are indicated with white Δ on all images. Scale bars on the BLI images show the minimum and maximum intensity in ph/s/cm²/sr. The images shown in this figure depict the most representative images of the study.

Supplementary Tables

Table S1. Affinity determination of sdAb 2BD42-s775z by Surface Plasmon Resonance. Mean and standard deviation of the association rate (k_a), dissociation rate (k_d) and affinity (K_D) of sdAb 2BD42 and the fluorescently labeled sdAb 2BD42-s775z. SPR was performed in triplicate.

	k_a (1/Ms)	k_d (1/s)	K_D (nM)
**sdAb 2BD42**	$1.77 \times 10^6 \pm 0.10 \times 10^6$	$2.26 \times 10^4 \pm 0.1 \times 10^4$	0.127 ± 0.008
**sdAb 2BD42-s775z**	$6.82 \times 10^5 \pm 0.21 \times 10^5$	$0.30 \times 10^4 \pm 0.01 \times 10^4$	0.440 ± 0.030
